# Two Venovenous Extracorporeal Membrane Oxygenation for One Gunshot

**DOI:** 10.1155/2022/1070830

**Published:** 2022-07-20

**Authors:** Louis Pot, Alizée Porto, Audrey Le Saux, Amandine Bichon, Emi Cauchois, Marc Gainnier, Julien Carvelli, Jeremy Bourenne

**Affiliations:** ^1^Réanimation des Urgences, Timone University Hospital APHM, 13005 Marseille, France; ^2^Cardiac Surgery Department, Timone University Hospital APHM, 13005 Marseille, France; ^3^Center for CardioVascular and Nutrition Research, INSERM, INRAE, Aix-Marseille University, 13005 Marseille, France

## Abstract

Venovenous extracorporeal membrane oxygenation (VV-ECMO) is an adjuvant treatment for severe acute respiratory distress syndrome (ARDS) with refractory hypoxemia. Contraindications to therapeutic anticoagulation must be ruled out prior to ECMO implementation. We report the case of a 17-year-old male admitted in intensive care unit (ICU) for penetrating chest trauma due to multiple gunshot wounds. The body computed tomography (body CT scan) documented right pulmonary contusions and a homolateral hemothorax. His condition rapidly deteriorated with refractory hypoxemia due to lung contusion requiring invasive mechanical ventilation (IMV) and polytransfused hemorrhagic shock. During his stay in ICU, venovenous ECMO (VV-ECMO) was implemented twice, firstly for trauma-induced ARDS and secondly after thoracic surgery. This case emphasizes the successful use of VV-ECMO in posttraumatic ARDS without increasing the risk of bleeding.

## 1. Introduction

VV-ECMO is now widely used in refractory ARDS. This recommendation particularly applies for medical causes of ARDS, as evidenced by several clinical trials [1]. VV-ECMO indication in posttraumatic refractory hypoxemia remains uncertain, especially in blunt chest trauma, and is sparsely used [2]. Moreover, there is no evidence of the benefic use of other adjuvant treatments in posttraumatic ARDS.

In this report, we highlighted our twice successful VV-ECMO experience in a patient admitted for pulmonary contusion and hemorrhagic shock due to multiple gunshot injuries, complicated with recurrent severe ARDS.

## 2. Case Report

We report the case of a 17-year-old male patient admitted in the emergency department with multiples gunshot injuries. The initial clinical assessment showed an oxygen saturation of 100% on 15 L/min of oxygen. Hemodynamic and neurological conditions were initially stable. The physical examination found bullet wounds on the right ankle, the left wrist, and the right thoracic wall (entry wound under the right axillary hollow). Another lesion on the left retro-scapular region could have corresponded to the exit wound.

Hemodynamic status rapidly deteriorated requiring massive transfusion with threshold values consistent with international neurotrauma guidelines (10 g/dL for hemoglobin and 100 G/L for platelets), volume expansion, and the introduction of norepinephrine.

The body computed tomography scan (CT scan) revealed an active contrast media extravasation within the right subscapular muscle and the right trapezium muscle, as well as a right pleural effusion. It also showed right pulmonary contusions associated with hemato-pneumatoceles of the right superior lobe. The bone window revealed a T6-T7 vertebral fracture with intraductal bone fragments in contact with the spinal cord.

The respiratory function secondarily deteriorated with oxygen saturation ranging between 85 and 92% on fraction of inspired oxygen (FiO2) of 1. Two intrapleural chest tubes were inserted, collecting a total of 1500 mL of hemothorax fluid. Upon refractory ARDS despite ventilation optimization and neuromuscular blocking agents (NMBA), a first VV-ECMO was implemented. Considering active bleeding, preclotted circuitry was used without systemic anticoagulation. The use of ECMO permitted an ultra-lung-protective ventilation with a FiO2 of 0.7, a positive end expiratory pressure (PEEP) of 16 cmH20, a tidal volume of 200 mL, and a respiratory rate of 9/min. The use of respiratory assistance with extracorporeal circulation led to an oxygenation improvement with ECMO parameters set to 3.8 L/min at a rate of 3,000 rpm and 4.5 L/min sweep gas flow with a fraction of delivered oxygen (FdO2) of 1.

The restoration of both respiratory and hemodynamic stability at day 1 after VV-ECMO implementation enabled the radiological embolization of three right intercostal arteries (K2K3K4) and the right superior thoracic artery. Heparin was then carefully introduced with ATTP objectives between 1.2 and 1.3 to minimize the hemorrhagic risk.

The evolution was favorable with significant hemodynamic improvement, resulting in the weaning of norepinephrine 48 hours after admission in ICU. The ultra-lung-protective ventilation was pursued with a FiO2 of 0.3. The PEEP was optimized at 12 cmH2O using dynamic transpulmonary driving pressure monitoring. Prone position was contraindicated in this case due to an untreated unstable spinal fracture.

NMBA were interrupted at day 3, and VV-ECMO was weaned at day 8 allowing the interruption of the anticoagulation therapy. Protective invasive mechanical ventilation was maintained. Heparin was reintroduced after 48 h upon a diagnosis of multiple ECMO-induced thromboses (right femoral and right internal jugular veins) revealed by the post-ECMO systematic CT scan.

At day 15, the patient's respiratory function brutally declined due to both recurrence of hemothorax (clogging in right chest tube) and ventilatory dysfunction, refractory to volume-controlled ventilation, accurate sedation, NMBA use, inhaled nitric oxide administration, and prone positioning which was used as a rescue therapy, considering the still untreated unstable vertebral fracture. Thus, a second VV-ECMO was initiated. The next day, thoracic surgery was needed to achieve hemostasis with a right upper partial lobectomy. Subsequent severe ARDS (FiO2 of 1, PEEP of 15 cmH20) with a major decrease of pulmonary compliance at 10 mL/cmH2O were observed. VV-ECMO parameters were adjusted to 4.5 l/min at a rate of 3,500 rpm, with a FdO2 of 1.

A lung biopsy of the right superior lobe revealed recent pulmonary fibrosis. Thus, a high-dose corticosteroid course (3 mg/kg/day) was initiated at day 24, leading to a progressive improvement of pulmonary compliance. The patient was switched to an airway pressure release ventilation (APRV) mode at day 28, and a percutaneous tracheostomy was performed the next day. VV-ECMO was weaned at day 30.

Mechanical ventilation was weaned at day 45 upon a significant respiratory improvement. A bronchoscopy was performed, showing a fistula of the right upper lobar bronchus with a positive blue-test. The tracheotomy was then removed, and the patient was discharged from the intensive care unit at day 60 after admission.

## 3. Discussion

Penetrating chest trauma is frequently complicated by hemorrhagic shock requiring immediate or delayed surgical intervention. The aims of surgery include intrathoracic lesions assessment, surgical hemostasis, and resection of necrotic tissue. In this context, anticoagulation is commonly contraindicated.

This case illustrates different issues regarding the use of VV-ECMO in posttraumatic ARDS. First, the initiation of ECMO in this case was an urgent decision taken immediately after admission, considering the severe hypoxemia despite optimized invasive mechanical ventilation. Indeed, the benefice-risk ratio was in favor of this therapy, but its use in this context is questionable. In our case, the use of ECMO was not associated with aggravated coagulopathy, but there is no evidence that trauma patients would benefit from this therapy without further bleeding complications. Thus, this success raises the question of the use of systemic anticoagulation during VV-ECMO, especially in patients with high bleeding risk.

We report here the first case of VV-ECMO reintroduction upon a second respiratory function deterioration in trauma-induced ARDS [3]. This therapy enabled a safe thoracic surgery achieving proper hemostasis, and the subsequent weaning from mechanical ventilation and lung recovery although the risks were considerable as the patient already had thrombotic complications induced by the first VV-ECMO.

Secondly, although widely used in ARDS for medical etiologies, VV-ECMO remains sparsely implemented in trauma patients [4]. Raff et al. showed in a national survey that only 45% of the responders reported using ECMO in adult trauma patients [2]. Although the main reason for not initiating this therapy was the lack of availability in their institution, many responders did not feel comfortable with the use of ECMO in trauma patients or considered that the bleeding risk was too high in this context.

Third, early continuous infusion of NMBA and prone positioning are recommended for nontraumatic moderate and severe ARDS [5, 6]. However, traumatic contexts, especially those with intracranial hypertension or unstable spinal, femoral, or pelvic fractures, were excluded from this algorithm [7]. In our case, the instable spinal fracture was an absolute contraindication for prone positioning leading to the decision of respiratory assistance by VV-ECMO. Thus, the current therapeutic algorithm regarding the management of moderate and severe ARDS is not accurate for trauma patients, especially for those presenting brain or spinal injuries [8]. Subsequently, VV-ECMO implementation holds a key position in this specific indication.

Posttraumatic ARDS presents several characteristics contraindicating the use of ECMO. Severe blunt chest trauma is usually not isolated, frequently associated with hemorrhagic shock or high bleeding risk situations. The use of extracorporeal circulation in this context increases the risk of complications, and this very particular indication must be performed with high volume ECMO in trauma center.

## 4. Conclusion

Prone positioning is contraindicated in trauma-induced instable spinal lesions. Subsequently, there is currently a gap in the algorithm of moderate-to-severe ARDS care. We described here the case of a successfully assisted patient with two successive VV-ECMO for recurrent severe ARDS and instable vertebral fractures preventing from prone positioning. Thus, a case-by-case VV-ECMO implementation could constitute a therapy of choice for these patients without increasing the risk of aggravated coagulopathy.
